# Thromboembolism in Patients with Hypertrophic Cardiomyopathy

**DOI:** 10.7150/ijms.50167

**Published:** 2021-01-01

**Authors:** Lu Liu, Zheng Liu, Xiaoping Chen, Sen He

**Affiliations:** 1 Department of Cardiology, West China Hospital of Sichuan University, Chengdu, China; 2 Nursing Department, West China School of Nursing, West China Hospital of Sichuan University, Chengdu, China; Lu Liu and Zheng Liu equally contribute to the article

**Keywords:** hypertrophic cardiomyopathy, thromboembolism, stroke, anticoagulant therapy, atrial fibrillation

## Abstract

Hypertrophic cardiomyopathy (HCM) is an inherited cardiac disease, which has a marked heterogeneity in clinical expression, natural history, and prognosis. HCM is associated with a high prevalence of thromboembolic events (stroke and systemic embolic events), even if taking no account of atrial fibrillation (AF), leading to unexpected disability and death in patients of all ages. Several risk factors of thromboembolism such as AF, greater age, left atrial diameter, heart failure and others have been confirmed in patients with HCM. Conventional thromboembolic predictive models were estimated by several trials in HCM population but it turned out to be unsatisfactory. Based on those previous explorations, researchers tried to modify or develop novel models suitable for HCM population in thromboembolism prediction. In consideration of catastrophic advent events of thromboembolism, current guidelines have recommended life-long anticoagulant therapy after a single short AF.

Therefore, early identification of risk factors for thromboembolism, accurate risk stratification, timely preventive measures and aggressive management may help to avoid serious adverse thromboembolic events in HCM population.

## Introduction

Hypertrophic cardiomyopathy (HCM) is characterized as hypertrophy of the left ventricle, usually asymmetric and involving interventricular septum^1^, ^2^. Myocyte hypertrophy and disarray as well as interstitial fibrosis are also the key pathological hallmarks. Moreover, impaired ventricular filling and dynamic left ventricular outflow tract (LVOT) obstruction are important pathophysiologic features^3^. It has been confirmed that HCM is an inherited disease, an autosomal dominant inheritance is a typical model^4^-^6^. Over 450 mutations in 20 sarcomeric and myofilament-related proteins have been identified, involving β-myosin heavy chain, cardiac troponin T, cardiac troponin I, α-tropomyosin, cardiac myosin binding protein C, the essential and regulatory myosin light chains, and cardiacactin^2^, ^7^-^10^. HCM is common with a prevalence of 1 in 500 and even up to 1 in 200^1^, ^11^-^15^. HCM is heterogeneous with a wide spectrum of clinical manifestations, including heart failure (HF), atrial and ventricular arrhythmias and sudden cardiac death, as well as thromboembolism^1^, ^11^, ^12^.

Thromboembolism events, including stroke and systemic embolic events, known as profound complications of HCM, have a high incidence rate in HCM patients and often lead to catastrophic adverse events, which is consequently related to increased morbidity and mortality^16^-^20^. For HCM patients, the current issues are mostly concentrated on the risk stratification of sudden cardiac death or the therapeutic dilemma of alcohol septal ablation versus surgical myectomy^21^. However, the high incidence and the poor prognosis of thromboembolic events in this population should not be ignored.

This review covers a series of issues concerning thromboembolic events in patients with HCM, with a focus on the epidemiology, risk factors, prediction models, and management.

## Epidemiology

According to previous studies all over the world, both the prevalence and incidence of thromboembolism are high in patients with HCM, especially in patients with atrial fibrillation (AF)^12^, ^13^, ^15^, ^22^, ^23^. In Asia, based on the data on patients diagnosed with HCM from the entire Korean population between 2005 and 2015, the prevalence of stroke was approximately 10%^24^. Similarly, in a regional Japanese cohort, the 5-year embolic event rate was 5.5%^25^. In Europe, a retrospective, longitudinal cohort of seven institutions, having excluded patients with a history of AF and thromboembolic events prior to the first evaluation, recruited 4821 HCM patients and demonstrated that 172 (3.6%) patients reached the primary endpoint within 10 years, including 105 cerebrovascular accidents (CVA), 53 transient ischaemic attacks (TIA) and 14 peripheral emboli, and the 5- and 10-year cumulative incidences were 2.9% and 6.4% (95% CI 2.37-3.48% vs. 5.42-7.53%, respectively)^26^.

The patients with HCM who have suffered thromboembolism have a poor prognosis. In a prospective trial, 900 patients with HCM were consecutively enrolled and prospectively followed between 1970 and 1998, and 51 (6%) patients suffered stroke or other vascular events over 7±7years, finally, 21 (41%) of these 51 patients died or were permanently disabled^20^. Recently, Lorenzini et al. reported that compared with the general population, adult patients with HCM had excess mortality in a large international multicenter referral cohort^27^.

## Risk Factors of Thromboembolism in HCM

### Atrial fibrillation

Atrial fibrillation (AF), determined by electrocardiogram, Holter monitoring, or medical history, is the most common sustained arrhythmia in HCM and has a prevalence of 20%-25% in HCM patients, which is 4-6 fold higher likelihood than that in the general population^13^, ^28^-^31^. The prevalence and annual incidence of thromboembolism events were 27.1% and 3.8% respectively in HCM patients with AF^13^, ^32^. In a study based on the entire Korean population between 2005 and 2015^24^, the prevalence of stroke in HCM patients with AF was 20%, double of that in the whole HCM patients. The authors also stated that during 8741 person-years, AF-related stroke occurred in 257 subjects among 2309 HCM patients with new-onset AF, and the overall incidence rate of AF-associated stroke was 2.94 per 100 person-years. In HCM patients, the incidence of ischaemic strokes was 8 times higher in patients with AF than in those with sinus rhythm^33^. This obvious discrepancy about thromboembolic events might be attributed to anti-cardiolipin antibody (aCLa), produced by some cell line in the HCM heart when AF occurs; another speculative possibility is that thrombogenesis of the endothelium in HCM hearts is enhanced by LVOT obstruction, though no assessment of this possibility is yet available^34^. HCM patients more commonly develop AF at a younger age in comparison with the general population^35^. AF is poorly tolerated by HCM populations, largely owing to their left ventricular filling dependent on atrial systole^13^, ^33^. The association between AF and high risk of thromboembolism in HCM patients has been confirmed^36^-^38^.

AF tends to be paroxysmal in two-thirds of HCM patients and persistent/permanent in the remaining one-third^33^. The definitions of different types of AF varied between studies^13^. Persistent AF was found in 50% of patients having thromboembolism^18^, while paroxysmal AF was reported in 11 out of 18 in the thromboembolic patients^39^. However, the incidence of thromboembolism between HCM patients with paroxysmal AF and those with chronic AF was reported not significantly different^33^, ^40^, and the thromboembolism risk in AF is not related to the number of paroxysms as well^41^.

### Left atrial diameter

Left atrial diameter (LAD) has also been verified to be a risk factor for thromboembolism, associated with the development of AF as well^42^. However, it has been found that the enlargement of the left atrial tended to be greater in the embolism group than that in those with AF but without systemic embolism^43^. In patients with HCM without AF, Haruki et al.^22^ have suggested that each1mmm increase in LAD prefigures the increased risk of stroke-related death (HR 1.10, 95% CI 1.00-1.20). As for the relationship between left atrial size and risk of thromboembolic events, there seems to be a linear relationship, and the risk of thromboembolism rises exponentially with increasing LAD up to 45-50mm^26^.

### Greater age

Greater age is also a significant risk factor acknowledged by a host of trials^20^, ^22^, ^26^, ^44^. Guttmann et al.^26^ developed exploratory multivariable analysis and revealed age as a predictor of thromboembolism in HCM (HR 1.03, 95%CI 1.02-1.04, p <0.001). Maron et al.^20^ stratified age and regarded age as independent (p<0.00). Moreover, the older the HCM patients were, the higher risks of thromboembolism they were faced with (RR 1.6, 95%CI 0.8-3.6 vs. RR 8.2, 95%CI 3.9-21.6 for 41-60 and >60 years old, respectively).

### Heart failure

Heart failure (HF), characterized by excessive exertional dyspnea, is a common complication with a broad illness states, from mild to severe (New York Heart Association [NYHA] functional classes II-IV) in HCM^45^, ^46^. In a prospectively followed-up of an HCM group^20^ , 51 patients experienced one or more cerebrovascular or other peripheral vascular events. And at the time of the initial event, 19 patients (37%) had severe symptoms (NYHA functional class III or IV), including 7 patients in the end-stage phase with systolic ventricular dysfunction. In a recent newly developed model predicting thromboembolic events for HCM patients, Cox regression revealed an association between thromboembolism and NYHA class, and the authors regarded congestive heart failure symptoms and NYHA functional class as risk factors^26^.

### Other risk factors

Late gadolinium enhanced (LGE) extent > 14.4% on cardiovascular magnetic resonance (CMR) was regarded as an independent predictor for thromboembolic events in HCM patients (sensitivity 65%, specificity 78.1%, AUC 0.79, P<0.0001)^47^. A study reported CHADS_2_ > 1 combined with left ventricular outflow tract gradient (LVOTG) >38 mmHg was an independent predictor of embolic stroke in HCM patients^48^. Additionally, some factors associated with AF like left ventricular outflow tract obstruction, systolic anterior movement (SAM), mitral regurgitation (MR)[49]predisposing hemodynamic might increase the thromboembolic risk. Others such as prior thromboembolism, maximal wall thickness, vascular diseases, and hyperuricemia have been put forward as risk factors on thromboembolism in HCM patients in some researches^20^, ^26^, ^50^, but the data is limited and further estimations are required.

According to the prior researches, several simple and common clinical features can be used to identify the risks of thromboembolism in HCM patients, which are concluded in Table 1.

## Thromboembolism Predictive Models

Regardless of the proposal of risk factors for thromboembolism, comprehensive risk stratification of thromboembolism in patients with HCM still challenges the clinical decision making and is urgent to be solved.

### HCM Risk-CVA

Not long ago, to determine the risk of thromboembolism in patients with HCM, a novel risk model called HCM risk for cerebrovascular accident (HCM Risk-CVA) was developed^26^. The risk factors identified by this model, based on a retrospective, longitudinal cohort of seven institutions, include (1) age; (2) presence of AF; (3) previous thromboembolism: ischemic stroke, TIA and peripheral embolism; (4) NYHA classes II-IV; (5) presence of congestive heart failure symptoms; (6) vascular disease: myocardial infarction, complex aortic plaque, and peripheral arterial disease; (7) left atrial diameter and maximal ventricular wall thickness (MWT). HCM Risk-CVA showed prominent discrimination (C-statistic 0.75, 95% CI 0.70-0.80; D-statistic 1.30, 95% CI 1.05-1.56) and a good calibration slope of 0.91 (95% CI 0.74-1.08) in the original study population.

The equation of the risk of thromboembolism in 5 years for an individual HCM patient is presented as following:

p = 1- 0.9999874^exp (prognostic index)^

where the prognostic index = 0.030417476 × age (years) + 2.129977874 × AF (yes = 1/no = 0) - 0.027069595 × age × AF + 1.288557829 × thromboembolic events prior (yes = 1/no = 0) + 0.224673046 × NYHA class II (yes = 1/no = 0) + 0.728180341 × NYHA class III/IV (yes = 1/no = 0) + 0.032251831 × left atrial diameter (mm) + 0.3735254 × maximal wall thickness (mm) - 0.008324216 × maximal wall thickness^2^ (mm) + 0.512492795 × vascular disease (yes = 1/no = 0).

Subsequently He et al.^51^ carried out an external validation of this model in a relatively large cohort of patients with HCM, although their calibration was not perfect, HCM Risk-CVA could still help to discriminate the risk of thromboembolism in the whole cohort and the subgroup without AF.

Nevertheless, the complexity of HCM Risk-CVA limits its wide application more or less^52^. HCM Risk-CVA may be considered to guide risk management among the HCM population and prospective external validations of HCM Risk-CVA in different distributions and different ethnic cohorts become the urgent need.

### CHADS_2_ and CHA_2_DS_2_-VASc

To predicting the thromboembolic events in patients with AF, the CHADS_2_^53^ and CHA_2_DS_2_-VASc^54^ were developed, and the CHA_2_DS_2_-VASc score was previously used as a means of stratifying patients with non-valvular AF for thromboembolic events prophylaxis^55^, ^56^. However, in the HCM population, current guidelines issued by the European Society of Cardiology (ESC) or Japanese Circulation Society (JCS) do not recommend those traditional scoring systems in risk stratifying of thromboembolism^49^, ^57^. Several literature validating those models have also pointed out the defects not correlating well with the clinical outcomes in patients with HCM^24^-^26^, ^58^-^61^. In HCM individuals with CHA_2_DS_2_-VASc scoring 0 or 1, the incidence rate of AF-associated stroke is 1.48 per 100 person-years^24^. According to a large-scale, longitudinal cohort study of HCM subjects in the absence of AF, those with CHA2DS2-VASc score ≤ 2 had a significantly increased risk of ischemic stroke, compared with matched general population with AF (score 0, HR 2.062, 95% CI 1.816 - 4.209, p = 0.026; score 1, HR 2.261, 95% CI 1.940 - 3.300, p = 0.018; score 2, HR 1.383, 95% CI 1.023 - 2.201, p = 0.034)^44^.

### Other Models

Tsuda et al.^61^ incorporated HCM into CHADS_2_ score and CHA_2_DS_2_-VASc score, the modified risk models were superior to the customary scores alone for predicting thromboembolism (P=0.0033 and P=0.0002, respectively). Furthermore, ROC curves under the Cox model was constructed, and the C-statistics for prediction of thromboembolism showed 0.7120 (95% CI 0.6675-0.7565), 0.7131 (95% CI 0.6697-0.7565), 0.7561 (95% CI 0.7133-0.7989) and 0.7675 (95% CI 0.7292-0.8059) for CHADS_2_ score, CHA_2_DS_2_-VASc score, CHADS_2_ score + HCM and CHA_2_DS_2_-VASc score + HCM, respectively.

R-CHA_2_DS_2_VASc score, which integrated GFR and BUN into CHA_2_DS_2_-VASc score and had been proved in good calibration and high discriminative performance predicting post-discharge ischemic stroke in a cohort of patients with a myocardial infarction before^62^, indicated a satisfied result in stratifying thromboembolic risk and was well calibrated in patients with HCM as well^63^.

## Thromboembolism Prophylaxis and Management

### Anticoagulant Therapy

Up to now, with CHADS_2_ and CHA_2_DS_2_-VASc not working well in the HCM population, there is no completely accordant scoring system available yet. In order to prevent thromboembolism as far as possible, ESC guidelines, ACCF/AHA guidelines, and several studies have strongly suggested the life-long oral anticoagulant therapy, warfarin therapy with goal international normalized ratio (INR) 2-3, in HCM patients with even a single brief episode of AF, regardless of the conventional stroke-scoring systems, particularly given the high likelihood of recurrence of AF and risk of thromboembolism. And the treatment should be ongoing even if sinus rhythm is restored^33^, ^49^, ^59^, ^60^, ^64^, ^65^.

In the absence of AF, thrombosis could be promoted as well, due to the abnormal atrial substrate^66^. Similarly, in HCM patients without documented AF, abnormal atrial substrate and function could increase the risk of thromboembolism once AF occurs. Based on previous studies, the overall burden of stroke in HCM was variable and not specified with AF^18^, ^19^. In a study enrolling 593 patients with clinically diagnosed HCM from 1980 to 2010^22^, the incidence of stroke and systemic embolic events was about 1.0% per year, and among the patients with events, however, there were more than half having not been documented AF before the embolic events. They also concluded that in patients with HCM without documented AF, older age and enlarged left atrial were possible risk factors for embolic events. Lin et al.^44^ identified 17371 HCM patients without AF and utilized propensity-score-matching to identify one-to-one matched control of the general population with AF receiving oral anticoagulants. During a median follow-up of 7.3 years, 847 (4.9%) subjects experienced ischemic stroke with the incidence of 0.589/100 person-years, and the corresponding matched controls experienced 788 (4.5%) events with the incidence of 0.494/100 person-years. Compared with control, the HCM group without AF had a similar risk of ischemic stroke (HRs 0.965, 95% CI 0.854-1.091), which implied that oral anticoagulants might be necessary for HCM patient even without AF. Stated thus, in terms of those HCM patients in the absence of AF but actually at thromboembolic risk (the older, LAD≥48mm, LGE>14.4% or others), prophylactic anticoagulant therapy might help prevent further thromboembolic events. At present, in clinical management, whether to apply prophylactic anticoagulant therapy remains to be ambiguous and urgent to be determined^22^, ^44^, ^47^, ^60^, ^67^.

Compared with those receiving warfarin, the cumulative incidence of stroke among patients with AF was significantly higher in the non-anticoagulant group (18% vs. 31%, p<0.05) in a consecutively enrolled and prospectively followed group of 900 HCM patients^20^. Another research also reported that embolic events were less common in patients taking anticoagulants (4/233, 2%) than in patients without anticoagulant prophylaxis (9/66, 14%, P<0.001)^39^.

Yet undertreatment in anticoagulation therapy exists. Real-world data from the GARFIELD-AF registry showed that the frequency of oral anticoagulants was less and target INR was lower in Asia than those in other regions of the world^68^. The actual use of oral anticoagulants in non-valvular atrial fibrillation (NVAF) patients with HCM was suboptimal that only 15.3% at the time of AF diagnosis and 61.8% throughout the study period received oral anticoagulants^37^. The situation in Europe is not optimistic as well that there are greater than one-third of patients with AF and known risk factors who should have received anticoagulation therapy not adhering to the prescription^69^.

New oral anticoagulants (NOACs) might be reasonable alternatives to vitamin K antagonists (VKAs), but there is a paucity of data to support their application in patients with HCM and AF 70. On the basis of current guidelines on HCM, if adjusted-dose VKAs fails to reach anticoagulant aims or if patients experience side effects of VKAs or are unable to undertake INR monitoring, a direct thrombin inhibitor (dabigatran) or an oral factor X_a_ inhibitor (e.g. rivaroxaban, apixaban) is recommended^49^, ^71^. From a system review, the use of NOACs was associated with a lower pooled incidence rate of thromboembolic events at 4.7% compared to 8.7% with VKAs, as well as a lower pooled incidence rate of major bleeding and all-cause mortality in the NOACs group^72^. A nationwide cohort study from the Korean National Health Insurance Service database reported that, in consideration of all-cause mortality (5.11 vs. 10.13 events per 100 person-years, respectively) and the composite of fatal cardiovascular events (0.77 vs.1.80 events per 100 person-years, respectively), NOACs are superior to warfarin^73^. The results mentioned above are generally consistent with those from other researches^74^, ^75^. Furthermore, a recent study found that NOACs are at least as safe and effective as VKAs in patients with HCM undergoing catheter ablation for AF^76^. Whereas, the safety and efficacy of NOACs in the HCM population needs to be investigated in large and prospective randomized controlled trials in the future. For paroxysmal or chronic AF, anticoagulation with vitamin K antagonist warfarin is recommended, although NOACs are available (eg, dabigatran or rivaroxaban)^77^.

### Antiplatelet Treatment

Besides the coagulation system, platelets can also be activated in patients with HCM^78^, ^79^. A study investigated the mean platelet volume (MPV), an indicator of platelet activation, and the result showed that the MPV could be elevated in patients with HCM^80^.

In contrast, a meta-analysis including 4052 patients with general AF from six randomized clinical trials showed that compared with aspirin, oral anticoagulants significantly reduced the likeliness to experience any stroke (2.4 vs. 4.5 events per 100 patient-years, HR 0.55, 95% CI 0.43-0.71), ischemic stroke (HR 0.48, 95% CI 0.37-0.63), or cardiovascular events (HR 0.71, 95% CI 0.59-0.85)^81^. Actually, some substantial evidence bases are in favor of anticoagulation therapy other than antiplatelet treatment in the respects of curative efficacy, safety, and side effects 82-84.

Thus, antiplatelet therapy using aspirin 75-100mg plus clopidogrel 75mg daily should be only considered when patients refuse the use of any oral anticoagulants (whether VKAs or NOACs)^49^.

### Other managements

Unless AF is recognized timely, the consequence frequently is left atrial thrombosis and embolization, leading to stroke^34^. Timely identification of asymptomatic AF means early anticoagulant therapy. An asymptomatic paroxysmal episode or a first paroxysmal episode of AF in patients without previously documented AF may lead to thromboembolism^85^-^88^. Clinically asymptomatic AF appears to be common in HCM, occurring in almost 25% of patients^89^, ^90^. The American guidelines recommend that monitoring via 24-hour ambulatory electrocardiogram for asymptomatic AF should be considered in subjects with HCM^71^. While the European guidelines seem to be more cautious that HCM patients who are in sinus rhythm, but with a left atrial anterior-posterior diameter ≥ 45mm should be monitored every 6 to 12 months via 48-hour ambulatory electrocardiogram^49^. Thus, HCM patients with otherwise considered cryptogenic stroke should be carefully monitored for AF since it was reported that 7.4% of them had new-onset AF at the time of thromboembolic events and 14.7% developed AF during evaluation after stroke^22^. Implantable loop recorders could be taken into consideration if it is necessary^87^.

Even though the development of AF in HCM patients may lead to poor clinical outcomes, there are no data specifically in HCM defining the relative benefits of rate versus rhythm control^31^, ^91^. Amiodarone may be a safe and effective option in clinical treatment for those with HCM with AF^15^, ^65^, ^92^. The use of amiodarone was reported associated with fewer embolic episodes in a small study of 52 HCM patients with AF^93^.

Catheter ablation (CA) may be another therapeutic choice in treating arrhythmia^49^, ^65^. But the effectiveness and safety of CA in HCM patients are controversial^94^, ^95^. The success rate of a single CA is lower in the HCM population due to AF recurrence, and patients in permanent AF had lower success rates than those in paroxysmal AF (50% vs. 77%, respectively)^96^. In general, left atrial dilation, mitral regurgitation, atrial fibrosis, and left outflow tract obstruction are also linked to lower success rates of CA^94^, ^97^. For the oral anticoagulant therapy after CA, a recent study of AF subjects pointed that in 522 AF patients receiving CA and then remaining in sinus rhythm, warfarin was discontinued in 79% of 256 patients without risk factors and in 68% of 266 patients with ≥ 1 risk factor. None of them had a thromboembolism event during 25 ± 8 months of follow-up, while patients older than 65 years or with a history of stroke were more likely to remain anticoagulated despite a successful outcome^98^. However, data on CA of AF in HCM patients are still sparse. Since highly recurrent AF after CA often required antiarrhythmic drugs or repeat ablation, continuing anticoagulation indefinitely in all patients after CA might be taken into account^39^ , and current guidelines also recommend life-long anticoagulation therapy even if the sinus rhythm is restored^49^. Generally, younger HCM patients with smaller atrial size, mild symptoms, and shorter duration of AF may have better clinical effects^99^. However, relevant data are still sparse. So whether younger HCM patients with paroxysmal AF who successfully managed by CA and maintained stable sinus rhythm should be treated lifelong with anticoagulation therapy needs further researches.

As for patients with obstructive HCM undergoing a surgical septal myectomy, a surgical Maze procedure can be an adjunctive operation for AF ablation, which has been proved to yield excellent hemodynamic benefits and may potentially decrease the risk of thromboembolic events^39^, ^100^-^102^.

According to a prospective randomized study, in adult patients with hypertrophic obstructive cardiomyopathy who have severe mitral regurgitation, mitral valve plasty contributed to a lower rate of thromboembolic events^103^.

## Conclusion

On balance, the HCM population is at increased risk of potentially catastrophic thromboembolic events. Some risk factors on thromboembolism in HCM, such as AF, greater age, LAD, HF, and others have been confirmed. Since CHADS_2_ and CHA_2_DS_2_-VASc worked poorly in patients with HCM, several novel predictive models such as HCM Risk-CVA have been developed for risk stratification and validated to be more effective in HCM individuals than the conventional ones. Nevertheless, these new models lack external validations and there is no generally accepted model to predict thromboembolism in those suffering HCM. Currently, the most essential therapeutic measure is the lifelong anticoagulation treatment in all patients who have experienced even a single short of AF. Other managements such as regular monitor via ambulatory electrocardiogram for those without diagnosed AF and amiodarone or radiofrequency catheter ablation for HCM patients with AF were previously reported associated with less embolic events.

### Prospect

In the HCM population, further data from large prospective studies regarding comprehensive thromboembolic risk factors, novel risk stratification models specialized for the HCM population with high sensitivity and specificity, and indications for prophylaxis, therapy, and prognosis are urgently needed.

## Figures and Tables

**Table 1:** Risk Factors of Thromboembolism in patients with Hypertrophic Cardiomyopathy

Risk Factor	Citation	P value	Strength	95% CI
Greater age	Guttmann et al. 26	<0.001	HR 1.03	1.02-1.04
	Haruki et al. 22	0.012	HR 1.03	1.01-1.06
	Maron et al. 20	<0.005	RR 1.6 ^a^ RR 8.2 ^b^	0.8-3.6 ^a^ 3.9-21.6 ^b^
	Lin et al. 44	<0.05	HR 1.278 ^c^ HR 1.757 ^d^	1.070-1.335 ^c^ 1.435-2.152 ^d^
AF	Guttmann et al.^26^	<0.001	HR 8.41	1.95-36.3
	Higashikawa et al.^19^	0.0001	RR 9.330	1.902-28.264
	Maron et al.^20^	<0.0001	RR 10.2	4.6-25.0
	Tian et al.^36^	0.03	HR 6.71	1.23-38.58
LAD	Haruki et al.^22^	0.016	HR 2.74	1.20-6.23
	Guttmann et al.^26^	<0.001	HR 1.03	1.01-1.05
	Tian et al.^36^	0.04	HR 1.10	1.00-1.20
Prior TE	Guttmann et al.^26^	<0.001	HR 3.63	1.81-7.29
	Wang et al.^50^	-	-	-
NYHA class Ⅲ,Ⅳ	Guttmann et al.^26^	<0.001	HR 2.07	1.35-3.17
	Maron et al.^20^	<0.05	RR 2.4	1.2-5.0
MWT	Guttmann et al.^26^	<0.001	HR 1.45	1.12-1.88
Vascular disease	Wang et al.^50^	-	-	-
Hyperuricemia	Wang et al.^50^	0.013	HR 2.67	1.24-5.76
LGE extent	Hohneck et al.^47^	<0.0001	OR 1.1	1.05-1.16

LAD = left atrial diameter; LGE = late gadolinium enhancement on cardiovascular magnetic resonance; MWT = maximal wall thickness; NYHA = New York Heart Association; TE = thromboembolic event; -, not given; ^a^, 41-60 years old; ^b^, over 60 years old; ^c^, 65-74 years old; ^d^, over 75 years old.

## Abbreviations

aCLa: anti-cardiolipin antibody
AF: atrial fibrillation
CA: catheter ablation
CMR: cardiovascular magnetic resonance
CVA: cerebrovascular accident
HCM: hypertrophic cardiomyopathy
HF: heart failure
INR: international normalized ratio
LAD: left atrial diameter
LGE: late gadolinium enhancement
LVOT: left ventricular outflow tract
MPV: mean platelet volume
MR: mitral regurgitation
MWT: maximal wall thickness
NOAC: new oral anticoagulant
NVAF: non-valvular atrial fibrillation
NYHA: New York Heart Association
SAM: systolic anterior movement
TE: thromboembolic event
TIA: transient ischaemic attacks
VKA: vitamin K antagonist
